# RNase P RNA from the Recently Evolved Plastid of *Paulinella* and from Algae

**DOI:** 10.3390/ijms151120859

**Published:** 2014-11-13

**Authors:** Pilar Bernal-Bayard, Leonor Puerto-Galán, Agustín Vioque

**Affiliations:** Instituto de Bioquímica Vegetal y Fotosíntesis, Universidad de Sevilla and CSIC, Américo Vespucio 49, 41092 Sevilla, Spain; E-Mails: pilar.bernal@ibvf.csic.es (P.B.-B.); leonor@ibvf.csic.es (L.P.-G.)

**Keywords:** chloroplast evolution, chromatophore, cyanobacteria, RNase P, tRNA processing

## Abstract

The RNase P RNA catalytic subunit (RPR) encoded in some plastids has been found to be functionally defective. The amoeba Paulinella chromatophora contains an organelle (chromatophore) that is derived from the recent endosymbiotic acquisition of a cyanobacterium, and therefore represents a model of the early steps in the acquisition of plastids. In contrast with plastid RPRs the chromatophore RPR retains functionality similar to the cyanobacterial enzyme. The chromatophore RPR sequence deviates from consensus at some positions but those changes allow optimal activity compared with mutated chromatophore RPR with the consensus sequence. We have analyzed additional RPR sequences identifiable in plastids and have found that it is present in all red algae and in several prasinophyte green algae. We have assayed *in vitro* a subset of the plastid RPRs not previously analyzed and confirm that these organelle RPRs lack RNase P activity *in vitro*.

## 1. Introduction

RNase P is an ubiquitous enzyme responsible for the generation of the 5'-end of tRNAs by a single endonucleolytic cleavage of 5'-extended precursors [1]. There are two fundamentally different types of RNase P. The first type discovered is a ribonucleoprotein with a catalytic RNA subunit [2], and is found in all bacteria and Archaea and in the nucleus of many eukaryotes. The catalytic RNA subunit (RPR, RNase P RNA) is conserved [3] but the number and nature of protein subunits (RPP, RNase P Protein) is variable, one protein in bacteria, 5 proteins in Archaea and 10 proteins in Eukarya nuclei [4]. The second RNase P type is a structurally unrelated RNase P that has been recently described and that is composed solely by protein, named PRORP (Proteinaceous RNase P). PRORP function was first described in human mitochondria [5] and later in plant nuclei and organelles [6,7], but its presence is widespread in eukaryotes, indicating an early origin in eukaryotes [8]. PRORP seems to have replaced the ancestral ribonucleoprotein enzyme in the organelles of several eukaryotic lineages and fully in plants [9] where an *rnpB* gene (encoding RPR) has not been identified, and a functional PRORP is present in all three cell compartments (nucleus, mitochondria, and chloroplast) [7]. The single protein PRORP can completely replace the RNA based multisubunit RNase P of *E. coli* [6] and yeast nucleus [10,11] without loss of viability, representing therefore an extreme case of convergent evolution.

The evolution of plastid RNase P is intriguing because many algae, like the Glaucophyte *Cyanophora paradoxa*, some Prasinophyte green algae and all red algae whose chloroplast genomes have been sequenced, encode an *rnpB* gene in their plastids genome [12] (see below). However, no protein subunit homologous to bacterial RPP has been identified in algae, except in the green algae *Ostreococcus tauri* [13]. Expression of the plastid RPR gene has been shown to occur in *C. paradoxa* [14] and in *Ostreococcus tauri* [13], although so far it has not been demonstrated the involvement of this RNA in RNase P activity *in vivo*. The plastid RPRs contain in general all the strictly conserved nucleotides and their predicted secondary structures are similar to the catalytic bacterial RPR structure. However the plastid RPRs have no RNase P catalytic activity [13,15], or extremely reduced activity *in vitro* [16]. In some cases, reconstitution of RNase P activity from plastid RPR and bacterial RPP has been shown [16,17]. It seems that plastid RPRs have lost catalytic proficiency either because they are more dependent on one or more unidentified protein subunits, or because a PRORP type enzyme has replaced its function. In fact, red and green algae encode PRORP [8] and in the case of the prasinophyte *Ostreococcus tauri*, PRORP has RNase P activity *in vitro* [13], although its cellular localization is not known. Interestingly, *O. tauri* also encodes a homologue to the bacterial protein subunit of RNase P (RPP) that can reconstitute RNase P activity *in vitro* with bacterial RPR but not with its own plastid encoded RPR [13]. Therefore, chloroplast evolution seems to result in reduced or no function of RPR and then complete loss of the *rnpB* gene in most green algae and plants.

The thecate amoeba *Paulinella chromatophora*, a member of the super group Rhizaria, contains two blue-green photosynthetic “chromatophores” that represent a recent acquisition (60 myr) [18] of a cyanobacterial endosymbiont, therefore independent from the single endosymbiotic event that gave rise to present day plastids some 1200 myr ago [19]. The chromatophore has all the hallmark traits of a true organelle: reduced genome [18,20], unable to grow independently of the host, and dependent on protein import from the host [21,22]. The gene content of the chromatophore is about one fourth the gene content of the closest cyanobacterial relative identified (*Synechococcus* WH5701). Several essential genes related to photosynthesis have been transferred to the host nucleus and their protein products are imported into the chromatophore [21,22]. Because of the recent acquisition of the chromatophore, it could provide new insight into different aspects of the process of how endosymbionts became organelles and on organelle evolution [20,23,24].

Our earlier work [15,17] had shown that the RNA encoded by the *rnpB* gene retained in some plastid genomes seems to be functionally defective. We have extended the study to additional plastid RPRs and have characterized the RPR from the chromatophore of two *P. chromatophora* strains to determine if in this independent, more recent evolving plastid, a similar process of RPR loss of function has happened.

## 2. Results and Discussion

**Figure 1:**
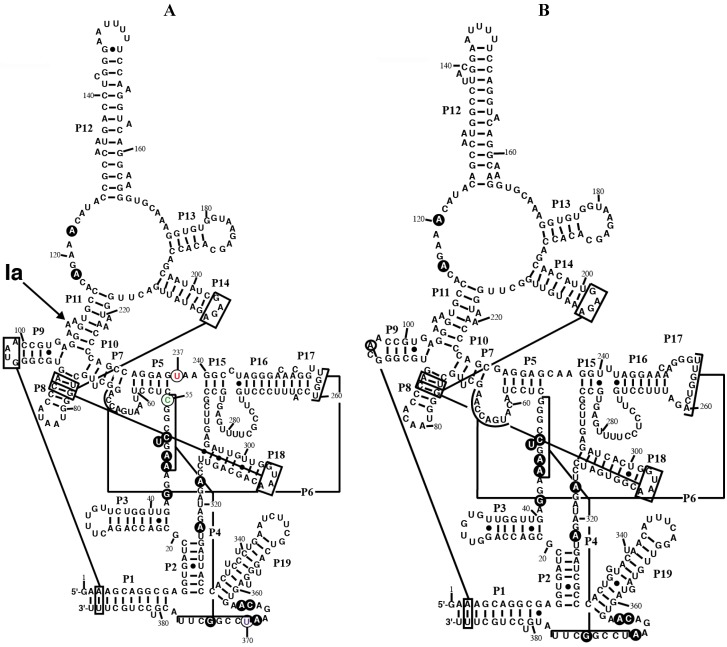
Secondary structure models of RPR from the chromatophore of *P. chromatophora*. The secondary structure models of RPR from the chromatophore of *P. chromatophora* M0880 (**A**) and *P. chromatophora* FK01 (**B**) are represented. Black circles highlight nucleotides universally conserved in bacteria [25]. Lines connect regions involved in tertiary interactions between conserved GNRA tetraloops and helices, as well as helices P4 and P6. The position of the main Pb^2+^-cleavage site (Ia) is indicated on the M0880 structure. Nucleotides C55 (green), U237 (red), and U370 (magenta), that deviate from the highly conserved consensus at these positions (see text) are circled.

The sequence of the chromatophore genome from *P. chromatophora* strain M0880 and *P. chromatophora* strain FK01 were searched for the highly conserved sequence of the P4 helix in RPR. The *rnpB* gene was unambiguously identified in both genomes. They contain all the residues universally conserved in bacteria [25]. Both RNAs can be folded into a secondary structure similar to the cyanobacterial RPR structure (Figure 1). However, some peculiarities could be observed. Helix P4 is one of the most conserved domains in RPR and is at the catalytic core of the ribozyme [26]. However we noticed that the last base pair of the conserved P4 helix in RPR from *P. chromatophora* M0880 (positions 55 and 370) is replaced by C-U. An inspection of 5800 bacterial RPR sequences present in the Rfam database [27] reveals that a canonical Watson-Crick base pair at this position is present in more than 96% of the sequences. A C-U pair is found in only 4 marine metagenome sequences, besides *P. chromatophora* M0880. What is more, position 237 has a highly conserved C (>94% conserved in bacteria, 100% conserved in cyanobacteria), but is a U in RPR from *P. chromatophora* strain M0880. The combination of C-U at the end of P4 and C at position 237 is unique for *P. chromatophora* M0880 RPR among the 5800 sequences analyzed. The sequence of RPR from strain FK01 is 86% identical to the sequence of RPR from strain M0880 and has a G-U pair at positions 55 and 370 (53 and 370 in the FK01 sequence). Position 237 (235 in FK01) is a C in the sequence of RPR from strain FK01, in agreement with the consensus.

**Figure 2:**
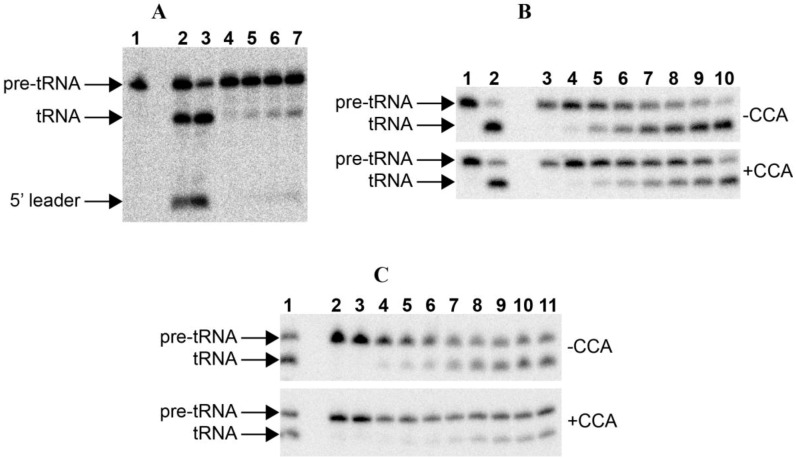
RNase P assays. (**A**) RNase P assay with *E. coli* pre-tRNA^Tyr^ of the RNAs alone: 1. blank without enzyme; 2–3. 50 nM *Anabaena* 7120 RPR incubated with the substrate for 30 and 90 min, respectively; 4–7. 50 nM *Paulinella* M0880 incubated with the substrate for 10, 30, 60 and 90 min, respectively. (**B**) RNase P assay with *Synechocystis* pre-tRNA^Gln^, either lacking or containing the 3'-CCA sequence, of the RNAs alone: 1. blank without enzyme; 2. 50 nM *Anabaena* 7120 RPR incubated with the substrate for 90 min; 3–10. 50 nM *Paulinella* M0880 RPR incubated with the substrate for 10 min, 30 min, 2 h, 4 h, 6 h, 8 h, 11 h and 24 h, respectively. (**C**) RNase P assay with *Synechcosytis* pre-tRNA^Gln^, either lacking or containing the 3'-CCA sequence, of the reconstituted holoenzyme with *Anabaena* RPP: 1. 50 nM *Anabaena* 7120 RPR reconstituted with *Anabaena* RPP and incubated with the substrate for 90 min; 2. control without RPP incubated with the substrate for 90 min; 3. control without RPR incubated with the substrate for 90 min; 4–11. 50 nM *Paulinella* M0880 RPR reconstituted with *Anabaena* RPP and incubated with the substrate for 5, 10, 15, 30, 60, 90, 120 and 180 min, respectively. The arrows indicate the position of the precursor tRNA, the mature tRNA and the 5'-leader fragment. The short 5'-leader in pretRNA^Gln^ runs out of the gel.

The *in vitro* transcribed RPRs from *P. chromatophora* M0880 was assayed for RNase P activity with an *E. coli* precursor tRNA^Tyr^ and a *Synechocystis* precursor tRNA^Gln^ (Figure 2). In both cases specific RNase P activity could be detected. The precursor tRNA was endonucleolitically cleaved at the same position with a control RNase P, generating fragments of the expected sizes.

**Figure 3:**
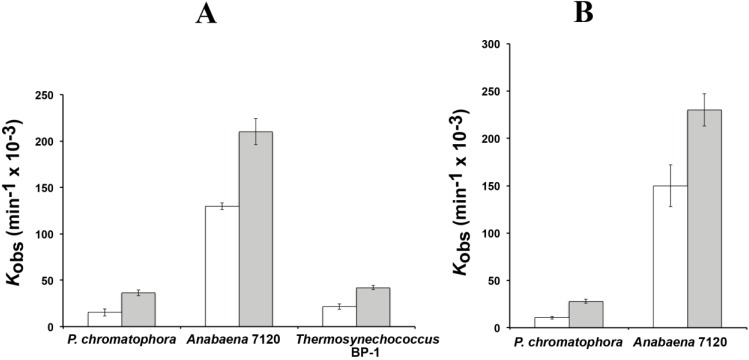
RNase P activity of RPR. Reaction rates were estimated with 0.05 µM of RPR from *P. chromatophora* M0880 and two cyanobacteria in the absence of protein (**A**) or in the presence of the purified RPP from *Anabaena* sp. PCC7120 (**B**). Assays were done as described in the Experimental Section with a precursor tRNA^Gln^ from *Synechocystis* sp. PCC6803 either containing (white) or lacking (gray) the 3' CCA sequence, under single turnover conditions. The average and standard deviation of three assays is represented.

The reaction rates of RPR from *P. chromatophora* M0880 and two different cyanobacteria, *Anabaena* 7120 and *Thermosynechococcus* BP-1, were analyzed for RNase P activity under single turnover conditions with two different versions of the *Synechocystis* pre-tRNA^Gln^ substrate, one containing the 3'-terminal CCA sequence and the other lacking this sequence (Figure 3). The 3'-terminal RCCA sequence interacts by base pairing with a conserved GGU sequence in the loop connecting helices P15 and P16 [28], and this interaction is an important determinant of cleavage efficiency and accuracy of bacterial RNase P [29]. However, cyanobacteria are an exception, and the presence of the CCA sequence is detrimental for activity. This anomaly has been related to the absence of conservation in sequence and size of the loop connecting P15 and P16 in RPR from cyanobacteria [30]. The CCA-lacking substrate was processed more efficiently by the chromatophore RPRs. The preference for CCA-lacking substrates is therefore a conserved property of chromatophore and free-living cyanobacteria. As found in cyanobacteria, *Paulinella* RPR lacks the conserved GGU sequence between P15 and P16 (Figure 1). The *P. chromatophora* M0880 RPR had a lower activity than *Anabaena* 7120 RPR but similar to *Thermosynechococcus* BP-1 RPR (Figure 3A) under RNA alone conditions. It could also reconstitute a functional holoenzyme with the *Anabaena* RPP (Figure 3B), although the heterologous reconstitution was significantly less efficient than the homologous reconstitution of *Anabaena* RNase P subunits. Bacterial RNase P holoenzyme has been reconstituted by mixing protein and RNA subunits of different origins. Although these heterologous reconstitutions are generally feasible, their efficiency is variable. Therefore we cannot draw quantitative conclusions about the relationship between the activity we measure *in vitro* and the endogenous holoenzyme.

In summary, our results demonstrate that *Paulinella* RPR has an *in vitro* ribozyme catalytic activity within the range of what is found for cyanobacteria and it can also reconstitute a functional holoenzyme with a heterologous cyanobacterial protein. This is in contrast with plastid RPRs, where only very week activity was observed with *Cyanophora paradoxa* RPR [16]. The *k*_obs_ described for *C. paradoxa* RPR was about 1 (min^−1^ × 10^−3^) at pH 6.0 [16]. It is difficult to compare with our results because our assays were done at pH 7.5 and with a different substrate.

**Figure 4:**
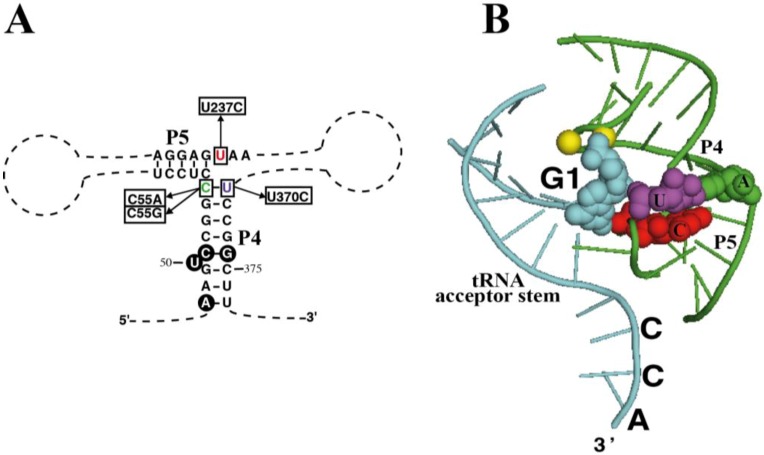
Mutagenesis of RPR. (**A**) Highlight of helices P4 and P5 in RPR from *P. chromatophora* M0880 indicating the nucleotides that were changed to generate the different mutant RPRs and (**B**) Crystal structure of the same RNA region from *Thermotoga maritima* RNase P-tRNA complex (PDB ID:3Q1Q) [26]. Part of the tRNA substrate acceptor stem is shown (cyan). Nucleotides 1 of the tRNA (G1, cyan) as well as the three nucleotides that were mutated are depicted as spheres with the same colors as in (**A**) and in Figure 1. In *T. maritima* RPR there is an A-U base pair at the end of P4, and position equivalent to the chromatophore U237 is the consensus C. The two magnesium ions at the active center are depicted as yellow spheres. The structure was rendered with Pymol.

As mentioned earlier, the chromatophore RPR has a C-U pair at the end of the highly conserved P4 helix (positions 55 and 370). What is more, nucleotide 237 is a U, instead of the highly conserved C at this position (Figure 4A). According to the crystal structure of bacterial RPR bound to tRNA (Figure 4B) [26], nucleotide 237 stacks on base pair 55-370, and they are very close to the active site, next to the first nucleotide of the tRNA and the two magnesium ions involved in catalysis. In order to assess the relevance of these otherwise conserved three nucleotides we have prepared several sequence variants of chromatophore RPR by site-directed mutagenesis and compared their RNase P activity *in vitro* under single turnover conditions with two different concentrations of RNA (0.05 and 4 µM) (Table 1).

**Table 1:** RNase P activity of wild type and mutant RPRs (RNA alone).

	[RPR] (µM)	*K*_obs_ (min^−1^ × 10^−3^) ^1^	Relative Activity (%) ^2^	P4 ^3^	237 ^4^	Count ^5^
WT (M0880)	0.05	36.5 ± 3.0	100	C-U	U	0
	4	300.8 ± 64.8	100			
C55A	0.05	1.7 ± 0.3	4.6	A-U	U	5
	4	21.0 ± 5.7	7.0			
C55G	0.05	1.2 ± 0.3	3.2	G-U	U	2
	4	15.5 ± 0.7	5.2			
U370C	0.05	11.8 ± 0.7	32.4	C-C	U	0
	4	246.7 ± 22.5	82.0			
C55G/U370C	0.05	2.3 ± 0.3	6.4	G-C	U	141
	4	38.8 ± 7.2	12.9			
U237C	0.05	6.5 ± 0.5	17.8	C-U	C	3
	4	nd				
C55A/U237C	0.05	9.8 ± 0.7	26.9	A-U	C	1140
	4	nd				
WT (FK01)	0.05	4.7 ± 0.3	12.8	G-U	C	62
	4	32.3 ± 1.3	10.7			

nd: not determined; ^1^ *K*_obs_ of the RPRs was determined in the absence of protein under single turnover conditions with two RPR concentrations. The average and standard deviation of three experiments is shown; ^2^ Relative activity is expressed as percentage of the activity of the wild type at the same enzyme concentration; ^3^ Sequence of base pair 55-370 (53-370 in FK01) of the different wild type and site directed mutants; ^4^ Sequence at position 237 (235 in FK01). The nucleotides modified in the mutants are in red. ^5^ Number of bacterial RPR sequences that contain the corresponding three-nucleotide combination out of the 5800 bacterial RPR sequences analyzed in the Rfam Database.

We first changed C55 for an A (C55A) or a G (C55G) to analyze how restoring a canonical A-U or G-U pair at the end of P4 affected activity. Surprisingly, the mutant RNAs had much lower activity than the wild type RPR from strain M0880, 7% and 5.2%, respectively with 4 µM RPR (Table 1). The double mutant C55G/U370C that has the preferred G-C base pair at the end of P4 had also reduced activity (12.9% with 4 µM RPR). However, when C replaced U370, generating a C-C pair, a combination that is present in only 0.3% of bacterial RPR sequences available, the activity was much higher (82% of wild type). Therefore it seems that the chromatophore RPR s optimized for a C at position 55, rather than for the presence of a canonical base pair at this position. Replacement of U237 by C, was also detrimental for activity, in spite the fact that a C is highly conserved at this position. U237C could partially rescue the deleterious effect of C55A mutation, indicating a functional interaction between both positions, in agreement with their close structural proximity (Figure 3B). In summary, it can be concluded that the chromatophore RPR has its overall structure optimized for the non-consensus combination of C55, U237 and U370. Similarly, a published attempt to restore or increase activity of the weakly active RPR from *Cyanophora paradoxa* by changing non-conserved nucleotides at otherwise highly conserved positions to the consensus sequence also resulted in the paradoxical loss of activity [16], suggesting that these divergent sequences are optimized in their overall structure, and the structure is perturbed in unpredictable ways when a specific position is modified. RPR from *Paulinella* strain FK01, that has a G-U base pair at the end of P4 had about 10% that activity of RPR from strain M0880.

Pb^2+^-induced cleavage is a useful probe of the tertiary folding of RNase P [31]. We have previously shown [15] that there is a significant difference in the Pb^2+^ cleavage pattern between cyanobacterial and plastid RPRs. The main cleavage site (Ia) in the three nucleotide bulge between helix P10 and helix P11 was absent in the plastid RPRs, pointing to a significant difference in the structure of this region, important for substrate interaction, that modifies divalent ion binding. We have probed the structure of *Paulinella* RPR by Pb^2+^ induced hydrolysis (data not shown). *Paulinella* RPR shows the main Pb^2+^ cleavage at site Ia (Figure 1), as in cyanobacteria [15]. There are no significant differences between the different mutants assayed for Pb^2+^-induced cleavage except for a slight reduction in Pb^2+^ sensitivity around position 140 in helix P12 in RNAs U370C and C44G/U370C. These results indicate that the analyzed mutations do not perturb the overall structure in a drastic way.

The *rnpB* gene has been previously described in several chloroplast genome sequences. We have done a comprehensive analysis of the growing collection of plastid genomes. Figure S1 and Table S1 present all the plastid *rnpB* sequences annotated in the databases up to date or identified by us as described in the Experimental Section. All available nineteen red algae chloroplast genomes contain an *rnpB* gene. In contrast, among the several hundred plant or green algae sequences available, the *rnpB* gene was identified only in five prasinophyte algae, an early branch in the green lineage of primary endosymbionts.

**Figure 5:**
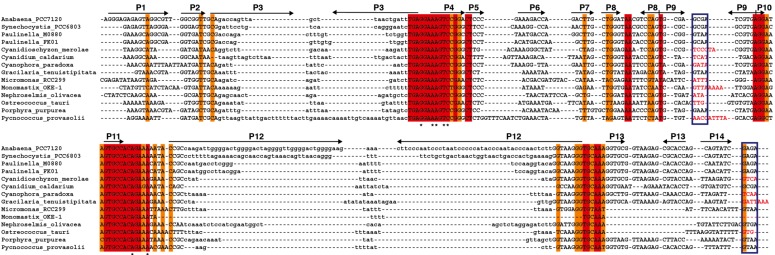

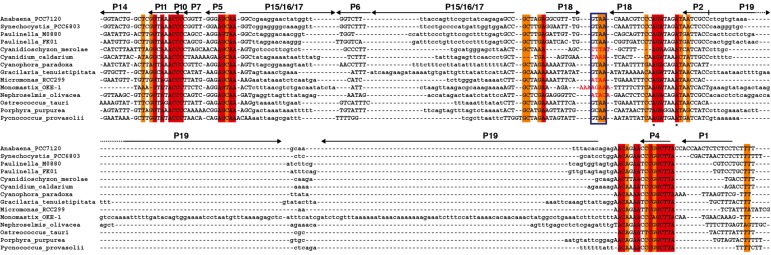
Alignment of *rnpB* genes. The *rnpB* sequence of selected cyanobacteria, *Paulinella*, and algae were aligned with clustalW [35] and the alignment was manually refined according to the secondary structures models of the different RNAs (Figure S2) with the help of Jalview [36]. The different helical segments are named P1 to P19 using the established nomenclature, as in Figure 1. Rightwards and leftwards arrows indicate the 5' side and the 3' side of a helix, respectively. The regions shown in lowercase, that correspond to helices P3, P12, P15–17, and P19 were not aligned due to the absence of sequence conservation. Red and orange shading indicate 100% and >80% sequence conservation, respectively. The blue boxes indicate the position of conserved GNRA tetraloops involved in tertiary interactions. Loop sequences that do not fit the GNRA tetraloop motif are in red. The universally conserved nucleotides are indicated by asterisks.

We have generated an alignment (Figure 5) and secondary structure models for several of the plastid RPRs (Figure S2). As with the previously described models for plastid RPRs, they fit the bacterial consensus, and contain the universally conserve nucleotides and the core conserved structure. However, except for *P. purpurea* RPR they all lack one or more of the conserved GNRA tetraloops present in stem-loops P9, P14, and P18. These tetraloops are important for stabilization of the tertiary structure of the RNA through long distance interactions between L9 and P1 [32], and between L14 and L18 with P8 [33] (Figure 1 and Figure S2). These tertiary interactions have been shown to be functionally relevant in *E. coli* [34]. Therefore the absence of intramolecular stabilizing tertiary interactions could be a possible explanation for the catalytic deficit of plastid RPRs (below).

Previous work had shown that the plastid RPR from *Cyanophora paradoxa*, *Nephroselmis olivacea*, *Porphyra purpurea*, and *Ostreococcus tauri* were inactive or very weakly active *in vitro* [13,15,16]. Here we have analyzed the RNase P activity of three additional red algae plastid RPRs (*Cyanidioschyzon merolae*, *Cyanidium caldarium*, and *Gracilaria tenuistipitata*) and two additional green algae plastid RPRs (*Micromonas* RCC299 and *Pycnococcus provasolii*).

For that purpose, the corresponding *rnpB* genes were cloned by PCR and used as templates for *in vitro* transcription with T7 RNA polymerase. The resulting RNAs were used in RNase P assays. We could not detect RNase P activity above unspecific degradation background with a number of different assay conditions (not shown). In addition to our standard assay conditions we tried high RNA concentration and long incubation times. We have also used the specific conditions described previously to detect catalytic activity of the human RPR [37], which is six orders of magnitudes lower than the activity of *E. coli* RPR.

These results confirm and generalize the apparent lack of RNase P activity of plastid RPRs and raise the question of what is the function of these RNAs if any *in vivo*, and why the *rnpB* genes are conserved in all red algae and several prasinophyte in spite of massive gene losses during plastid evolution. One possibility is that they are actually responsible for the essential RNase P function in the chloroplast but are more strictly dependent on one or more unidentified protein subunits, so that their activity cannot be revealed *in vitro* in the absence of those protein cofactors. This missing protein cofactor would be expected to be a protein homologue to bacterial RPP. However a *rnpA* gene encoding this hypothetical protein is not found in the available sequence information from algae, with the exception of the *rnpA* gene identified in *Ostreococcus* and closely related prasinophytes, whose function is unknown [13]. Another possibility is that that plastid *rnpB* genes are non-functional relics (pseudogenes) and the plastid RNase P function is provided by a PRORP type of enzyme, similarly to the situation in higher plant chloroplasts. In fact, a functional PRORP was identified in *O. tauri* [13], although it is not known if the protein localizes to the chloroplast. Sequences with the active site signatures of PRORP (PPR and ribonuclease NYN motifs) are found in green algae and in the red algae genomes [6]. However the available data strongly suggest a functionality for *rnpB* genes in algae: firstly, in *C. paradoxa* and *O. tauri* the plastid *rnpB* genes have been shown to be expressed [13,14]; secondly, *rnpB* is present in all known red algae chloroplast genomes sequenced without exception, that sample the whole evolutionary radiation of this highly diverse group, and finally, the plastid RPRs contain all the universally conserved nucleotides despite the low overall conservation of sequence and structural elements, suggesting a functional constraint on their sequence. The plastid expression of *rnpB*, the strict conservation of gene presence, and nucleotide conservation argue against the hypothesis that plastid *rnpB* are pseudogenes. A variety of non-tRNA substrates have been described for bacterial RNase P, such as the precursors of 4.5S RNA [38], phage RNAs [39], some mRNAs [40,41], and riboswitches [42,43]. Likewise, RPR might be retained in those plastids that have a functional PRORP for some additional function, independent of tRNA processing, such as processing of specific mRNAs or non-coding RNA substrates different from tRNAs. Finally, it cannot be excluded that plastid RPRs have acquired a completely novel unknown function (exaptation).

## 3. Experimental Section

### 3.1. rnpB Gene Identification

The plastid DNA sequences where *rnpB* had not been previously annotated were searched by Blast [44] with the highly conserved AAGTCCG sequence, which corresponds to the 5'-half of the universally conserved helix P4. The list of hits was manually inspected for the presence of the 3'-half of P4 at the expected distance (200–400 bp) downstream the hits. Sequences were aligned with clustal Omega [35] and the alignment was manually refined (Figure 5) according to the secondary structures models of the different RNAs (Figure S2) with the help of Jalview [36].

### 3.2. Cloning of RPR

Total genomic DNA from *P. chromatophora* strain M0880 or from *P. chromatophora* strain FK01 was used as template to amplify the *rnpB* gene of both strains with oligonucleotide pairs PchrRPR_F and PchrRPR_R (strain M0880) or PchrRPR_F and PFK01RPR_R (strain FK01) (Table S2) and cloned in pUC19 for *in vitro* transcription. The forward primer contains a T7 phage RNA polymerase promoter upstream the 5'-end of the *rnpB* gene. The reverse primer contains a *Dra*I restriction site just downstream the 3'-end of the *rnpB* gene. Different point mutations in the *rnpB* gene of *P. chromatophora* strain M0880 were generated with the QuickChange Site-Directed Mutagenesis Kit (Stratagene) using oligonucleotide pairs containing the sequence change desired (Table S2).

The *rnpB* gene of the cyanobacterium *Thermosymechococcus elongatus* BP-1 was cloned in the same way with oligonucleotides Telo_F1 and Telo_R1 (Table S2). The cloning and *in vitro* transcription of *Anabaena* sp. PCC7120 has already been described [30].

The *rnpB* genes from *Cyanidium caldarium*, *Cyanidioschyzon merolae*, *Gracilaria tenuistipitata*, *Micromonas* sp. RCC299, and *Pycnococcus provasolii* were cloned in pUC19 for *in vitro* transcription by PCR as above using the oligonucleotide pairs indicated in Table S2.

### 3.3. RNase P Assays

RNAs were prepared by *in vitro* transcription with T7 RNA polymerase (Promega) of *Dra*I digested plasmid and RNase P activity was measured under single turnover conditions as described [30], The precursor tRNA substrates used were a precursor tRNA^Tyr^ from *E. coli* [45] wit a 43 nucleotides 5' extension and a precursor tRNA^Gln^ from *Synechocystis* sp. PCC 6803 with a 10 nucleotides 5' extension. Two variants of the pretRNA^Gln^ were used, one containing the 3' CCA sequence (pretRNA^Gln^CCA) and the other lacking it (pretRNA^Gln^) [30]. The substrates were uniformly labeled by *in vitro* transcription in the presence of [γ-^32^P]CTP. The plastid RPRs assays were performed at pH 6.0 as described [37].

Recombinant His-tagged RPP from *Anabaena* sp. PCC 7120 was purified as described from overexpressing *E. coli* cells [46] and used in holoenzyme reconstitution assays as described [30].

### 3.4. Pb^2+^ Cleavage Assays

Pb^2+^-induced cleavage sensitivity analysis was carried out as described [15] with RPRs labeled at the 5'-end with [γ-^32^P]ATP and T4 polynucleotide kinase, after dephosphorylation with calf intestinal phosphatase.

## 4. Conclusions

We have studied *in vitro* the RNase P activity of RPR present in the chromatophore of *Paulinella*, a model of the early evolution of chloroplasts and find it to be functional, in contrast to plastid RPR. Some nucleotides that deviate from the conserved consensus are optimized for the chromatophore RPR function. *rnpB* is present in all red algae and in prasinophyte green algae but no catalytic activity could be demonstrated *in vitro* for the encoded RPR. The function, if any, of the plastid RPR remains to be characterized.

## Supplementary Materials

Supplementary materials can be found at: http://www.mdpi.com/1422-0067/15/11/20859/s1.
